# 48, XXXY/49, XXXXY mosaic: new neuroradiological features in an ultra-rare syndrome

**DOI:** 10.1186/s13052-015-0156-0

**Published:** 2015-07-14

**Authors:** Donatella Milani, Francesca Bonarrigo, Sabrina Avignone, Fabio Triulzi, Susanna Esposito

**Affiliations:** Pediatric Highly Intensive Care Unit, Department of Pathophysiology and Transplantation, Università degli studi di Milano, Fondazione IRCCS Ca’ Granda Ospedale Maggiore Policlinico, Via Commenda 9, 20122 Milan, Italy; Neuroradiology Unit, Fondazione IRCCS Ca’ Granda Ospedale Maggiore Policlinico, Milan, Italy

**Keywords:** Cranio-cervical junction, Klinefelter syndrome, Fraccaro syndrome, Sex chromosomal aneuploidies, X chromosome

## Abstract

**Background:**

Sex chromosomal aneuploidies in males are rare diseases with an overwhelming involvement of endocrinological and auxological issues; less frequently, other anomalies are observed. Neuroradiological issues are often not taken into account in single patients, and neuroradiological examinations are rarely performed.

**Case presentation:**

Here, we report a boy with 48,XXXY/49,XXXXY mosaicism, phenotypically characterized by hypotonia, intellectual disability, ventricular septal defect, micropenis, and with mild hypertelorism, inverted nipples, a congenital hip dysplasia, and some neuroradiological features so far not described. The Magnetic Resonance Imaging showed white matter abnormalities and enlargement of lateral ventricles with never described dysmorphisms of cranio-cervical junction and posterior fossa. A cranio-cervical Computerized Tomography confirmed a dysmorphic aspect of the posterior fossa and occipital condyles, slight morphological asymmetry of C1 and slight lateralization to the right of the odontoid’s apex.

**Conclusions:**

Considering the possible relevant clinical impact of these findings, the neuroradiological assessment seems potentially useful to the diagnostic approach of these patients.

## Background

Genetic conditions characterized by a variation in sex chromosome’s number are defined as sex chromosomal aneuploidies (SCAs). The most common of them is Klinefelter syndrome (KS). About 80 % of male patients with an X chromosome gain has the classical 47,XXY karyotype, whereas 20 % has other SCAs (48,XXXY, 48,XXYY, 49,XXXXY), a chromosomal mosaicism, or other structurally abnormal sex chromosomes [1].

KS has a prevalence of 1 in 500 males; its clinical features include infertility, gynecomastia, eunuchoidism, small testes and penis and hypergonadotropic hypogonadism. 48,XXXY syndrome has an incidence of 1:50,000, whereas 49, XXXXY (also known as Fraccaro syndrome) is the rarest one, with an incidence of 1:85.000–1:100.000 male births [1].

48, XXXY, 49, XXXXY and KS share some phenotypic traits, but trisomy and tetrasomy of the X chromosome show a different phenotype with more severe clinical features [2, 3].

The height of patients with 48,XXXY syndrome as well as those with KS is above the average, particularly after puberty. This feature is not found in 49,XXXXY syndrome where height is below the average; this condition is often described as the most severe variant of the spectrum. Clinical features include characteristic facial appearance, intellectual disability, hypogonadism, severe speech delay, multiple skeletal anomalies, and cardiac defects [1, 3].

SCA patients have lower intellectual quotient (IQ), related with the number of X chromosomes; every additional X chromosome reduces IQ by about 15 points [4]. The social, cognitive, and neurological development of patients with SCA are highly variable [1, 3, 4]. Patients with 48,XXXY have language difficulties, social and emotional problems [1, 4]. Patients with 49,XXXXY have lower IQs, nonverbal and verbal production difficulties [1, 4]. Usually, they are shy but sociable, although they also easily go wild with outbursts of anger if frustrated [1, 3, 4].

Brain magnetic resonance imaging (MRI) studies on KS have provided evidence that sex-chromosome polysomy exerts specific effects on brain development [5]. Individuals with XXX and XXY can have smaller brain volumes (microencephaly), enlarged lateral ventricles, cortical atrophy, hypoplastic or thin corpus callosum, and white matter abnormalities [4, 5]. Brain MRI in 48, XXXY showed nonspecific white matter hyperintensities. 49,XXXXY is associated with more markedly decreased brain volume and increased incidence of white matter hyperintensities [1].

We describe a patient with a 48,XXXY/49,XXXXY mosaicism having some unusual and undescribed neuroradiological features.

## Case presentation

**Fig. 1:**
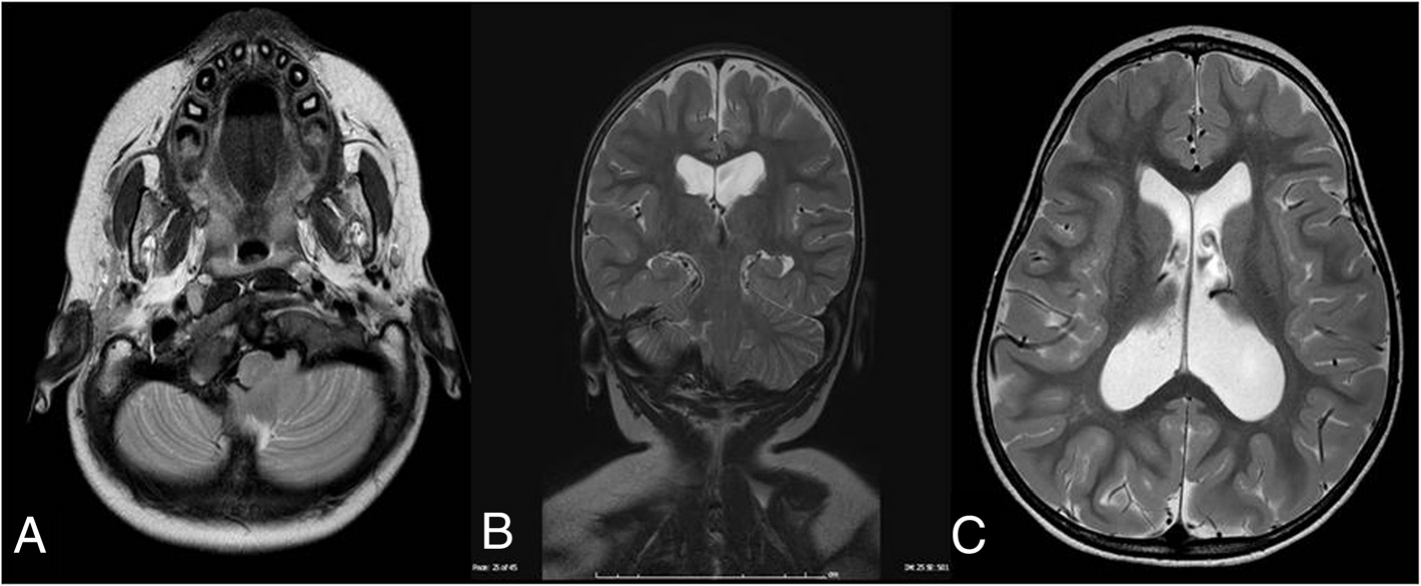
**a** and **b** Axial and coronal T2 images showing asymmetric and dysmorphic posterior fossa, right rotation and asymmetry of C1-C2 as well as reduced visualization of cerebrospinal fluid spaces. **c** Axial T2 image showing ventriculomegaly and small focal hyperintensities of the frontal and parietal white matter

**Fig. 2:**
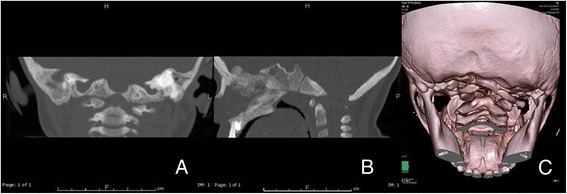
**a** Coronal CT multiplanar reconstruction showing asymmetric occipital condyles aspects. **b** Sagittal CT multiplanar reconstruction shows clivus platybasic aspect. **c** 3D reconstruction (bone volume rendering) showing dysmorphic aspects of cranio-cervical junction

The patient is a 20-months-old boy born to non-consanguineous parents at 39 weeks of gestational age by iterative caesarean section, which followed a normal pregnancy. His birth weight was 2.650 Kg, birth length 46 cm, occipitofrontal circumference (OFC) 34.5 cm, Apgar 10/10. At birth, he presented facial dysmorphysms, inverted nipples, and ventricular septal defect (VSD). The cerebral ultrasonography showed a slight hemorrhage of the germinal zones. Furthermore, a congenital hip dysplasia was diagnosed. He came to our attention for developmental delay, congenital heart defect and hypogenitalism. Weight was 10.2 kg (10th percentile), length 81 cm (25th percentile) and OFC 48 cm (25–50th percentile). He had mild hypertelorism, flattened nasal bridge, slightly pronounced chin, inverted right nipple, small penis, small hands and mild generalized hypotonia. Due to his phenotype, our geneticist (DM) recommended to perform a karyotype analysis; the analysis showed a mosaic karyotype 49,XXXXY[12]/48,XXXY[8]. Considering this cytogenetic result and according to the literature, an X-ray examination of arms was performed and this ruled out radioulnar synostosis. Moreover, a brain MRI was performed. A moderate ventriculomegaly with predominant expansion of the posterior segments and slight asymmetry was found. Small focal hyperintensities of the frontal, parietal and posterior periventricular white matter were detected; they are associated with a mild reduction of white matter. The myelination was normal for his age. There were no alterations of the cerebellar and brainstem signal. The MRI showed an asymmetric dysmorphic appearance of the posterior cranial fossa, small in size, with dysmorphism of cranio-cervical junction (CVJ) and reduced visualization of cerebrospinal fluid spaces (Fig. 1). A cranio-cervical Computerized Tomography (CT) was performed to better assess the anomalies of the skull base and it confirmed a dysmorphic aspect of the posterior fossa and occipital condyles, slight morphological asymmetry of C1, regular morphology of C2 and slight lateralization to the right of the odontoid’s apex (Fig. 2).

## Conclusions

49,XXXXY is a very rare condition often classified as a KS variant; however, various authors proposed that it should be diagnosed as an independent clinical syndrome [2]. KS variants may present different features, medical problems and complications. Our case includes some of these (i.e., dysmorphisms and hip dysplasia) [6]. Gropman et al. described patients with 48,XXXY syndrome with tics, intentional tremor and generalized hypotonia [7]. At present, only a mild generalized hypotonia emerged in our patient.

Brain abnormalities are frequently found in KS variants; in particular, loss of brain volume, ventriculomegaly, white matter hyperintensities (such as T2 hyperintensity signals ranging from extensive confluent white matter alterations to punctate foci), decreased width of the corpus callosum, and cortical atrophy are reported [5, 7]. Therefore, the supernumerary X chromosome seems to have a negative effect on white matter and central nervous system (CNS) development [4]. Gropman et al. correlated these findings with the neurodevelopemental performance, mainly in 49,XXXXY patients [4].

As a matter of fact, the brain MRI performed on our patient showed ventriculomegaly, white matter hyperintensities, particularly of the frontal and parietal lobes, and anomalies of posterior fossa and CVJ. Raven et al. reported a Chiari type 1 malformation associated with ventriculomegaly and a cervical syrinx in a patient with 49,XXXXY syndrome; this was found after performing a brain MRI on the patient after his first episode of seizure [8]. Chiari malformation can be associated with cranio-cervical junction anomaly, but in our patient, we found an isolated CVJ dysmorphism, better highlighted by CT that confirmed the asymmetric and dysmorphic aspect of the posterior fossa. We found a platybasic and asymmetric aspect of clivus with elongated right portion towards omolateral occipital condyle that was hypertrophic and prominent, whereas the left occipital condyle was hypotrophic as the left clivus portion. C2 and odontoid’s apex had normal morphology even if there was a slight lateralization to the right of the apex.

To the best of our knowledge, our patient is the first case of a CVJ malformation associated with 48,XXXY/49,XXXXY syndrome. Some intriguing considerations about a possible genotype-phenotype correlation can be drawn. In particular, we suggest that the mosaicism found in our patient can lead to a milder neuroradiological phenotype compared to Raven’s patient. Mosaicism could also have an effect on two different embryogenetic components of CVJ (the central pillar and the surrounding rings) leading to different and coexisting dysmorphic aspects (platybasia and hyper-hypoplastic occipital condyles). Therefore, it is important to underline that skeletal abnormalities are not exclusively related to the limbs, but also to the axial structures (such as the cervical spine) and could give neurological signs. Considering all the reported abnormalities and the possible relevant clinical impact of some findings, the neuroradiological assessment (MRI and CT) seems potentially useful in the diagnostic approach to patients with 48,XXXY and 49,XXXXY syndrome.

## Consent

This case report has been approved by the Ethics Committee of Fondazione IRCCS Ca’ Granda Ospedale Maggiore Policlinico, Milan, Italy. Written informed consent was obtained from the patient's parents forpublication of this Case report and any accompanying images. A copy of the written consent is available forreview by the Editor-in-Chief of this journal.

## Abbreviations

CT: Computerized tomography
CNS: Central nervous system
CVJ: Cranio-cervical junction
IQ: Intelligence quotient
KS: Klinefelter syndrome
MRI: Magnetic resonance imaging
OFC: Occipitofrontal circumference
SCA: Sex chromosomal aneuploidies
VSD: Ventricular septal defect
